# Study on dark septate endophytic (DSE) fungi and soil nutrients of *Ulmus pumila* L. in sandy land in eastern Inner Mongolia

**DOI:** 10.3389/fmicb.2026.1782229

**Published:** 2026-04-13

**Authors:** Yunxia Ma, Yuexin Zhang, Guang Yang, Yazhou Shao, Xiaolu Ma, Haibing Wang

**Affiliations:** 1 State Key Laboratory of Water Engineering Ecology and Environment in Arid Area, Inner Mongolia Agricultural University, Hohhot, China; 2 College of Desert Control Science and Engineering, Inner Mongolia Agricultural University, Hohhot, China; 3 Department of Ecology and Meteorology, College of Forestry, Inner Mongolia Agricultural University, Hohhot, China

**Keywords:** community structure, dark septate endophytic fungi, sandy land, soil nutrients, *Ulmus pumila* L.

## Abstract

Dark septate endophytic (DSE) fungi are widespread root-colonizing endophytes that play significant roles in host plant growth and adaptation. *Ulmus pumila* L., as a climax species in sparse forest-grassland ecosystems of sandy regions, contributes critically to regional ecological stability. In this study, root samples were collected from nine plots across three major sandy lands in Inner Mongolia—the Hulunbuir, Horqin, and Hunshandake Sandy Lands—to isolate and characterize DSE fungi. By combining traditional morphological classification with molecular identification, we determined the species composition, community structure, and spatial distribution of DSE fungi in *U. pumila* roots. Concurrently, key rhizosphere soil nutrient indicators were measured, and their relationships with DSE community distribution were analyzed to identify environmental drivers of DSE community assembly. A total of 200 DSE strains were isolated, which were classified into 16 species across 12 genera, 11 of which were identified to species level. Pure culture colonies exhibited diverse morphologies, predominantly black, dark green, or brown in color. DSE community composition differed markedly among sampling locations, with dominant species varying across sites. Pairwise similarity coefficients were all below 0.33, indicating substantial spatial heterogeneity. The Saihanwula site exhibited the highest colonization rate (22%). Soil total phosphorus (TP), total nitrogen (TN), and organic matter (OM) were identified as principal factors influencing DSE colonization rates, while soil moisture content (SMC), TN, and OM were key determinants of DSE community evenness. These findings provide a foundation for understanding root-associated DSE diversity in *U. pumila* and their ecological relationships with soil nutrient patterns in sandy ecosystems.

## 1 Introduction

*Ulmus pumila* L. (Ulmaceae) is a deciduous tree naturally distributed across the Hunshandake, Hulunbuir, and Horqin Sandy Lands of Inner Mongolia (Zhang et al., 2025). In 2021, it was listed as Critically Endangered (CR) by the International Union for Conservation of Nature (IUCN) and is recognized as a key species for combating desertification. *U. pumila* possesses a well-developed root system and exhibits high drought and cold tolerance, with strong adaptability to soils of low to moderate fertility. It plays a vital role in environmental protection, water conservation, windbreak, and sand fixation. By mitigating wind erosion and reducing dust–airflow interactions, it provides suitable habitats for fauna and facilitates the establishment of other plant species, making it an ideal candidate for afforestation in semi-arid sandy regions (Feng et al., 2014; Huang et al., 2023). Additionally, as a resource tree species, its leaves, bark, and roots contain numerous bioactive compounds and trace elements, offering potential sources of health-promoting foods and medicinal products (Lee et al., 2021; Chen and Xu, 2012; Park et al., 2016; Lykholat et al., 2018). *U. pumila* is widely distributed in eastern Inner Mongolia and constitutes a dominant vegetation type in the region’s sandy landscapes, where sparse *U. pumila* forests form the climax community. However, due to unplanned development and overgrazing, these ecosystems have experienced varying degrees of degradation across the three sandy lands. Consequently, the conservation and restoration of *U. pumila* sparse forest grasslands, alongside efforts to combat severe grassland desertification, are urgent ecological priorities. Among potential restoration strategies, the use of beneficial root-associated symbiotic fungi has recently been recognized as an effective approach to enhance plant environmental adaptability. In particular, dark septate endophytic (DSE) fungi play crucial roles in promoting host stress tolerance and nutrient acquisition in harsh environments (Santos et al., 2021; Malicka et al., 2022). Nevertheless, the composition and ecological roles of DSE communities associated with *U. pumila* in sandy lands remain largely unexplored.

Rhizosphere microorganisms, which rely primarily on root-derived nutrients, engage in complex interactions with plants that significantly influence host growth, development, and yield, while also contributing to soil health (Avis et al., 2008; Thepbandit and Athinuwat, 2024; Hakim et al., 2021; Ren et al., 2023). DSE fungi represent a major group of root-colonizing endophytes and, together with arbuscular mycorrhizal fungi (AMF) and ectomycorrhizal fungi (EMF), comprise the principal groups of root-associated symbiotic fungi. These groups differ substantially in morphology and ecological function. AMF (phylum Glomeromycota) possess aseptate hyphae and form arbuscular structures that primarily facilitate host phosphorus uptake. EMF (mainly Basidiomycota and Ascomycota) develop a fungal mantle on root surfaces and a Hartig net between cortical cells, predominantly associating with woody plants. In contrast, DSE fungi constitute a functional-morphological group encompassing diverse taxa, predominantly Ascomycota, and are characterized by dark, septate hyphae and microsclerotia within roots. Melanin deposition in DSE hyphae confers enhanced tolerance to environmental stress. In arid and nutrient-poor sandy habitats, DSE often outcompete AMF due to multiple advantages: melanin strengthens hyphal walls, protects against UV radiation, reduces oxidative damage, and aids water retention; microsclerotia act as dormant structures, enabling long-term survival under adverse conditions; additionally, DSE can be cultured on artificial media, facilitating inoculum development, whereas AMF are obligate biotrophs that cannot be grown axenically. Consequently, in drought-prone and nutrient-limited sandy land ecosystems, DSE may assume more critical ecological roles than AMF.

DSE fungi exhibit broad host compatibility and ecological distribution, maintaining high colonization rates even in extreme environments such as drought-stressed, metal-contaminated, or saline-alkaline soils. To date, DSE strains from over 100 families and 320 genera have been isolated from more than 600 plant species (Jumpponen et al., 1998). For instance, Han et al. (2021) reported 12 genera and 10 species of DSE isolated from 25 medicinal plant species, with soil organic carbon and pH identified as significant drivers of DSE diversity. Functionally, DSE fungi enhance host plant fitness by promoting mineral nutrient uptake, facilitating organic nitrogen assimilation, and increasing tolerance to abiotic (e.g., drought, heavy metals) and biotic stresses (Harsonowati et al., 2020; Farias et al., 2020; Malicka et al., 2022). He et al. (2021) demonstrated that DSE inoculation under drought stress increased biomass and bioactive compound concentrations in *Glycyrrhiza uralensis*. Additionally, Spagnoletti et al. (2017) and Barresi et al. (2022) showed that DSE fungi solubilize recalcitrant Ca-P and Al-P, thereby enhancing plant-available phosphorus and improving P uptake by *Sorghum bicolor*.

Despite their recognized ecological significance, most DSE studies have focused on herbaceous plants and shrubs under controlled stress conditions, leaving a notable knowledge gap concerning tall trees in natural, desertification-prone ecosystems such as sparse forest grasslands (Xu et al., 2024; Lu et al., 2025; Wang et al., 2024). The rhizosphere microbiome, particularly DSE fungi, likely plays a vital role in enhancing stress resistance and nutrient acquisition in *U. pumila*, yet this remains poorly understood. Previous research on *U. pumila* has primarily addressed community ecology, physiological stress responses, and hydrology (Cho et al., 2016; Dulamsuren et al., 2009; Su et al., 2014; Xia et al., 2024), with limited attention to root-associated fungal symbionts. Leveraging such beneficial plant–microbe interactions represents a promising and ecologically sustainable strategy for the restoration of degraded vegetation.

Based on this research background, the present study addressed the following questions: (1) What are the species composition, community structure, and spatial distribution of DSE fungi associated with *U. pumila* roots across the three major sandy lands of eastern Inner Mongolia? (2) How do soil physicochemical properties and enzyme activities influence DSE community colonization and diversity? We hypothesized that DSE community composition in *U. pumila* roots exhibits significant spatial heterogeneity among different sandy lands, and that soil total phosphorus (TP), total nitrogen (TN), and organic matter (OM) act as key environmental drivers of DSE distribution. To test this hypothesis, we aimed to: (1) isolate and characterize DSE fungi colonizing *U. pumila* roots across major sandy lands in eastern Inner Mongolia; and (2) analyze DSE species diversity, spatial distribution patterns, and key soil physicochemical factors influencing community assembly. By integrating field sampling with morphological and molecular identification, this study provides foundational data on cryptic fungal symbionts of this keystone tree species and offers a scientific basis for incorporating microbial tools into *U. pumila* conservation and sandy land ecosystem restoration.

## 2 Materials and methods

### 2.1 Study sites

**Figure 1:**
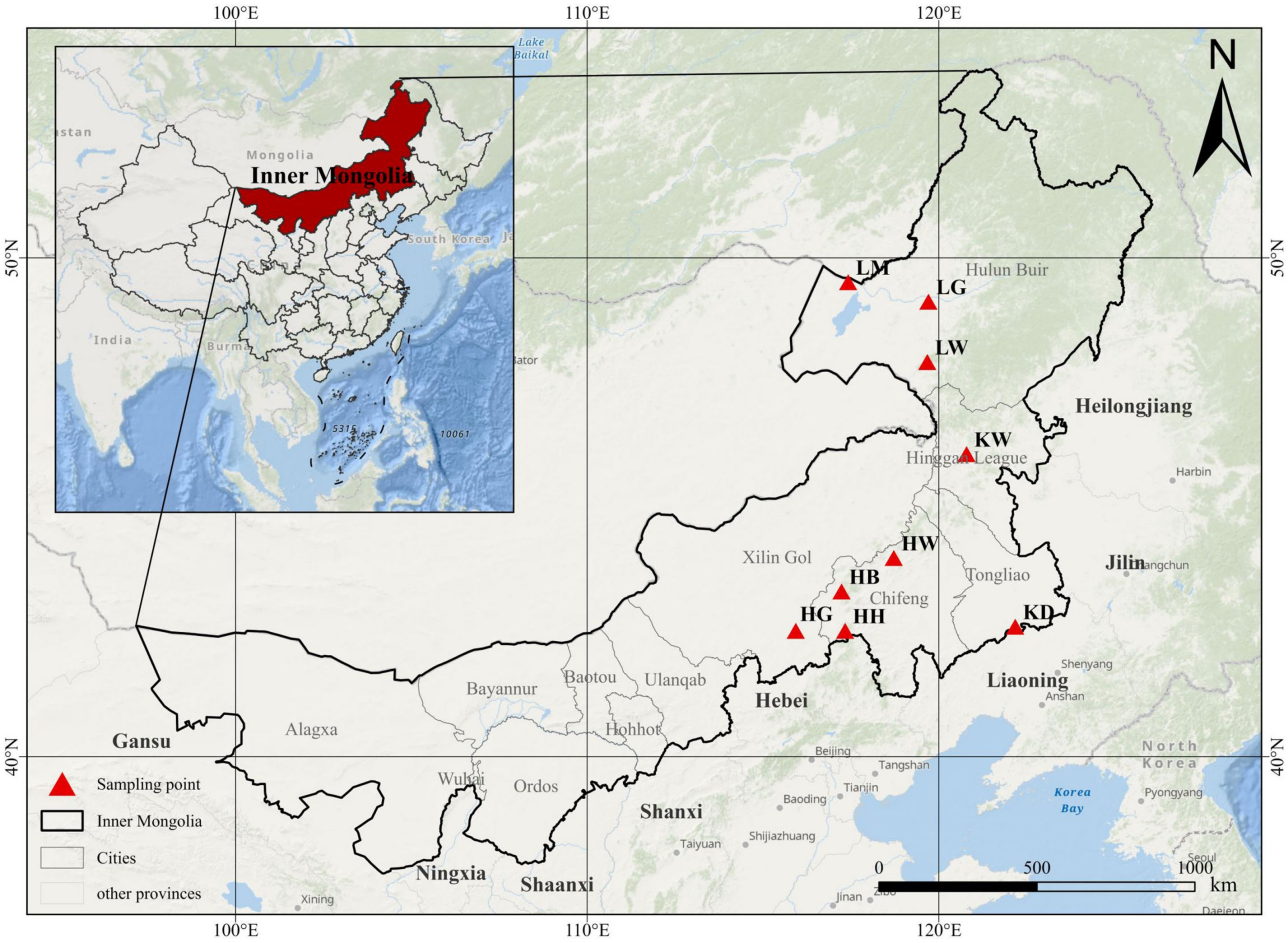
Schematic diagram of sampling points. HB: Baiyin Aobao; HH: Huamugou; HW: Saihanwula, HG: Sangendalai; KD: Daqinggou; KW: Wulanmaodu; LW: Wubulbaolige, LM: Manzhouli (LM), LG: Hailar.

Three major sandy lands are located in eastern Inner Mongolia: the Hunshandake, Horqin, and Hulunbeier Sandy Lands. The *Ulmus pumila* sparse forest grassland is the dominant vegetation type in all three regions and represents the climax ecological community. Accordingly, sampling was conducted within the *U. pumila* sparse forest grasslands of these sandy lands. A total of nine sampling areas were selected: Baiyin Aobao (HB), Huamugou (HH), Saihanwula (HW), and Sangendalai (HG) in Hunshandake Sandy Land; Daqinggou (KD) and Wulanmaodu (KW) in Horqin Sandy Land; and Wubulbaolige (LW), Manzhouli (LM), and Hailar (LG) in Hulunbuir Sandy Land (Figure 1, Supplementary Table S1).

Based on soil properties, the nine sampling plots were grouped for comparative analyses: (1) high versus low soil organic matter (SOM) plots, with SOM content >0.7 g/kg defined as high and <0.2 g/kg as low; (2) high versus low soil moisture plots, with soil water content (SMC) > 20% defined as high and <5% as low. These classification thresholds were determined according to the overall distribution of soil properties in the study area and were used to examine DSE community structure under contrasting environmental conditions.

### 2.2 Soil and root sampling

Sampling was conducted in August 2024, during the peak growing season. All nine sites were located on sunny slopes. To ensure comparability, forest stands with similar site conditions—including afforestation history, altitude, slope aspect, and slope gradient—were selected. Within each sampling area, nine plots were established, with a minimum distance of 100 m between any two plots to ensure spatial independence. From each plot, three replicate root and rhizosphere soil samples were collected from individual *U. pumila* trees. Fine roots (diameter <2 mm) were excavated aseptically and placed in sterile bags. Rhizosphere soil was defined as the soil remaining tightly adhered to the root surface after gently shaking off loosely attached soil, and was collected by brushing the root surface with a sterile brush. In the laboratory, each root sample was divided into two portions: one portion was stored at 4 °C for subsequent DSE isolation and purification, and the other was fixed in FAA (Formalin-Aceto-Alcohol) solution for microscopic observation of DSE colonization structures. Soil samples were air-dried in a well-ventilated, shaded area, sieved through a 2-mm mesh to remove litter and gravel, and stored for subsequent analysis of physicochemical properties.

### 2.3 Isolation of root-colonizing DSE

Fresh, asymptomatic root samples stored at 4 °C were randomly selected for fungal isolation. Adhering soil was gently removed by rinsing the roots under tap water through a 1-mm sieve. Root segments were then surface-sterilized in a laminar flow hood under UV light for 10 min, followed by immersion in 75% (v/v) ethanol for approximately 10 min. Subsequently, roots were rinsed three to five times with sterile distilled water and blotted dry on sterile filter paper. Using sterilized scalpels and forceps, roots were aseptically cut into ~0.5-cm segments, which were placed on potato dextrose agar (PDA) plates. Plates were incubated in darkness at 25 °C for up to 20 days, with daily monitoring of fungal growth. Emerging colonies exhibiting dark, septate hyphae characteristic of DSE fungi were subcultured onto fresh PDA plates supplemented with antibiotics (e.g., 50 mg/L chloramphenicol) to obtain pure cultures.

### 2.4 Molecular identification of DSE

Mycelium from actively growing DSE colonies was used for genomic DNA extraction using the AG21009 universal genomic DNA extraction kit (Acori). The internal transcribed spacer (ITS) region was amplified by polymerase chain reaction (PCR) using primers ITS1 (5′-TCCGTAGGTGAACCTGCGG-3′) and ITS4 (5′-TCCTCCGCTTATTGATATGC-3′). The 20 μL PCR reaction mixture consisted of 2.0 μL DNA template, 10 μL 2× Taq Master Mix, 1.0 μL of each primer, and 6.0 μL double-distilled water (ddH2O). PCR cycling conditions were as follows: initial denaturation at 95 °C for 4 min; 37 cycles of 94 °C for 30 s, annealing at 54 °C for 1 min, and extension at 72 °C for 1 min; followed by a final extension at 72 °C for 10 min.

PCR products were purified and sequenced by Sangon Biotech (Shanghai) Co., Ltd. Complete ITS sequences of the DSE strains were submitted to the NCBI database. Sequences were first compared against GenBank using BLAST,^1^ and phylogenetic analyses were conducted in MEGA (Version 11.0, Mega Limited, Auckland, New Zealand) using maximum likelihood tree construction.

### 2.5 DSE colonization and diversity analysis

The isolation frequency (IF) for each DSE strain was calculated as the number of occurrences of that strain divided by the total number of fungal isolates (Sun et al., 2011). The colonization rate was calculated as the percentage of root segments from which DSE were successfully isolated relative to the total number of root segments processed. DSE diversity was assessed using the Shannon-Wiener index (H), dominance using the Simpson index (D), and evenness using the Evenness index (E) (Wang et al., 2025). The following formulae were applied:


$$\begin{array}{l} \text{IF}=(\text{Occurrences of}\;a\;\text{specific}\;\mathrm{DSE}\;\text{strain})\\ /(\text{Total number of fungal isolates}) \end{array}$$ (1)

$$\begin{array}{l} \text{Colonization rate}\;(\%)\\ =(\begin{array}{l} \text{Number of root segments with}\;\mathrm{DSE}\;\text{isolation}\\ /\text{Total number of root segments processed} \end{array})\times 100 \end{array}$$ (2)

$$H=-\sum_{i=1}^{s}q_{i}\log q_{i}$$ (3)

$$D=\sum q_{i}^{2}$$ (4)

where ‘*q_i_’* is the colonization frequency of each DSE.


$$E=H^{’}/\ln S$$ (5)

where “*S*” is the total number of fungi isolated.

These indices capture different aspects of DSE community structure. The Shannon index integrates species richness and evenness, is sensitive to low- to medium-abundance taxa, and reflects overall community diversity. The Simpson index is sensitive to dominant species and indicates the degree of community dominance. The Evenness index, independent of species richness, characterizes the uniformity of species distribution. Combined use of all three indices provides a comprehensive characterization of DSE community diversity patterns.

### 2.6 Soil nutrient determination

Soil pH was measured in a 1:2.5 (w/v) soil-to-water suspension using a pH meter (Mettler Toledo FE20). Soil moisture content (SMC) was determined gravimetrically by oven-drying fresh soil at 105 °C to a constant weight. Available phosphorus (AP) was extracted with 0.5 M NaHCO₃ (pH 8.5) and quantified using the molybdenum blue method. Available potassium (AK) was determined via NH₄OAC extraction followed by flame photometry. Alkali-hydrolyzable nitrogen (AN) was measured using the alkaline hydrolysis diffusion method. SOM content was estimated using the potassium dichromate (K₂Cr₂O₇) oxidation method (Walkley-Black method). TP, TN, and total potassium (TK) were analyzed using a Smartchem 200 discrete autoanalyzer (Alliance, France) (Bao, 2000).

Soil enzyme activities were determined as follows: urease (Ure) activity was measured based on ammonium released from urea incubation using the indophenol blue method; sucrase (S-SC) activity was determined from reducing sugars released during sucrose incubation; acid phosphatase (S-ACP) and alkaline phosphatase (S-AKP) activities were quantified based on p-nitrophenol released from p-nitrophenyl phosphate disodium substrate. All enzymatic assays were conducted using commercially available kits (Beijing Solaibao Technology Co., Ltd., China), following the manufacturer’s protocols.

### 2.7 Statistical analysis

Statistical analyses were performed using SPSS 26.0 (IBM Corp., USA). Differences in soil physicochemical properties, DSE colonization, community composition, and diversity among plots were evaluated using one-way analysis of variance (ANOVA). *Post hoc* comparisons were conducted with Tukey’s test and Student’s *T*-test, with significance set at *p* < 0.05. Diversity indices of DSE communities were calculated using the vegan and ggplot2 packages in R (version 4.3.0). Mantel tests were performed to assess the effects of soil properties on DSE isolation rate and community diversity using the ‘linkET’ package. Partial least squares path modeling (PLS-PM) was conducted using the ‘plspm’ package in R. Values presented in figures represent the mean of at least three replicates.

## 3 Results

### 3.1 DSE isolation culture and morphological characteristics

**Figure 2:**
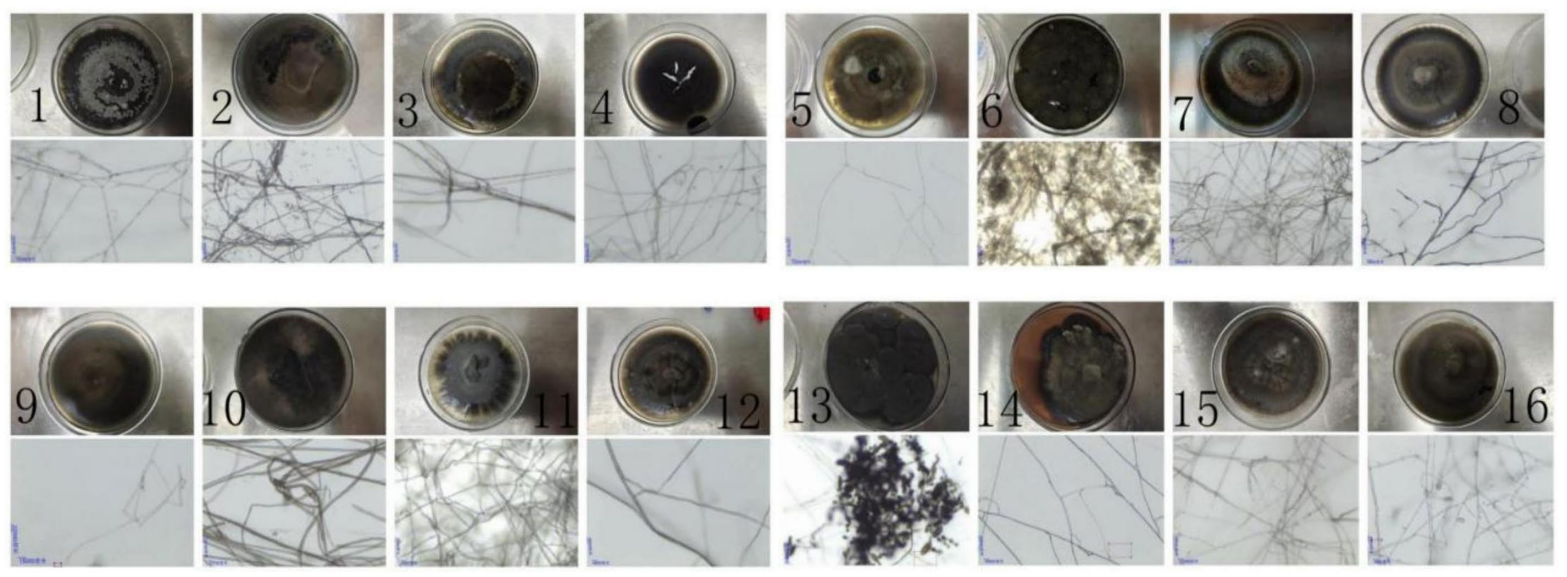
Colony morphology and microscopic morphology (scale plate = 20 μm). 1, HLE3; 2, DQG2; 3, SHWL6; 4, MZL1; 5, BYAB4; 6, HMG4; 7, DQG5; 8, DQG3; 9, WLMD6; 10, DQG4; 11, WBEBLG3; 12, MZL7; 13, BYAB3; 14, BYAB4; 15, HMG3; 16, SHWL6.

As shown in Figure 1, Supplementary Table S2, 16 DSE strains were isolated from the nine sampling areas and formed distinct colonies on PDA medium. Colonies exhibited unique macroscopic characteristics, including size, surface and reverse coloration, surface topography, texture, elevation, and margin morphology. Representative colony morphologies are presented in Figure 2. Microscopic features also varied among strains. The number of DSE species identified per sampling area was as follows: KW (7), LW (5), KD (3), LM (10), LG (6), HW (3), HG (3), HH (7), and HB (6). Notably, colony pigmentation generally intensified with prolonged incubation.

### 3.2 DSE molecular characteristics

ITS rDNA sequences were successfully obtained from representative colonies of each DSE morphotype, with fragment lengths ranging from 525 to 617 bp. BLASTn analysis against the NCBI GenBank database revealed that the 16 DSE strains isolated from *U. pumila* rhizosphere shared 97–99% sequence similarity with their closest reference strains. Phylogenetic analysis classified these isolates into 12 genera: *Cladosporium*, *Phialophora*, *Paraphoma*, *Phoma*, *Phialocephala*, *Cadophora*, *Lophiostoma*, *Alternaria*, *Stagonospora*, *Cylindrocarpon*, *Pteridiospora*, and *Phomopsis* (Table 1).

**Table 1:** Partial ITS sequence alignment of DSE strains isolated from *Ulmus pumila* roots.

Site	Strain ID	Reference taxon (accession number)	Sequence similarity (%)
HB	BYAB3	*Alternaria alternata* (KF293886.1)	100
	BYAB4	*Alternaria tenuissima* (FJ949086.1)	99
	BYAB1	*Phialocephala fortinii.* (KY910210.1)	99
HH	HMG4	*Cladosporium cladosporioides* (MF327241.1)	99
	HMG3	*Cadophora orchidicola* (MG062775.1)	99
HW	SHWL6	*Phialocephala* sp. (JQ088276.1)	98
HG	SGDL2	*Phialophora mustea* (JX406520.1)	99
KD	DQG5	*Cylindrocarpon* sp. *strain h8* (KY910192.1)	100
	DQG3	*Lophiostoma* cf. *cynaroidis A33* (JX434669.1)	99
	DQG4	*Phomopsis* sp. (KT270037.1)	100
KW	WLMD6	*Phoma herbarum* (EU823313.1)	99
LW	WBEBLG7	*Cladosporium tenuissimum* (HM776419.1)	99
	WBEBLG3	*Pteridiospora spinosispora* (MH859360.1)	99
LM	MZL7	*Stagonospora bicolor* (MH300001.1)	99
LG	HL3	*Paraphoma chrysanthemicola* (MH063747.1)	100
	HL1	*Cadophora* sp. BESC103j (KC007139.1)	100

**Figure 3:**
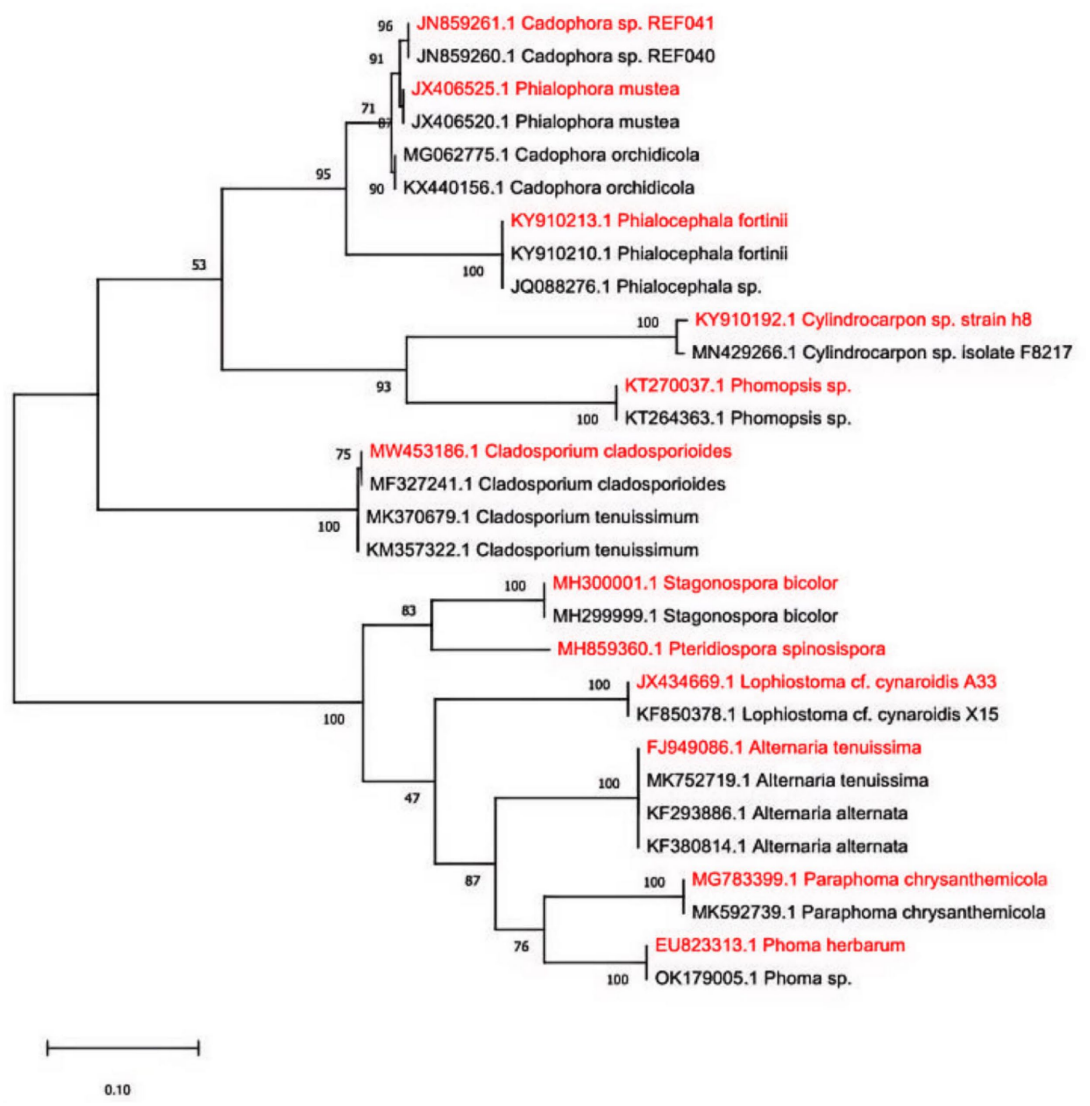
Maximum likelihood (ML) phylogenetic trees constructed from DSE strains isolated from *U. pumila* root systems across nine sampling areas. Branch bootstrap values represent the credibility of the corresponding phylogenetic relationships. Bold labels indicate strains isolated in this study. These strains belong to two major clades: Clade I: *Cadophora, Phialophora, Phialocephala, Cylindrocarpon, Phomopsis, Cladosporium*; Clade II: *Lophiostoma, Stagonospora, Alternaria, Pteridiospora, Phoma, Paraphoma*. Scale bar indicates distance = 10% nucleotide diversity.

Figure 3 presents a maximum likelihood phylogenetic tree constructed from these sequences along with reference sequences from NCBI. The isolates were divided into two major clades: Clade I comprised *Cadophora*, *Phialophora*, *Phialocephala*, *Cylindrocarpon*, *Phomopsis*, and *Cladosporium*; Clade II included *Lophiostoma*, *Stagonospora*, *Alternaria*, *Pteridiospora*, *Phoma*, and *Paraphoma*. Within Clade I, *Cadophora* and *Phialophora* formed a highly supported subclade closely related to *Phialocephala*, while *Cylindrocarpon* and *Phomopsis* grouped into a distinct monophyletic subclade, and *Cladosporium* was relatively distant. In Clade II, *Stagonospora* and *Pteridiospora* clustered on a single branch, separate from other taxa. *Phoma* and *Paraphoma* formed a subclade, together with *Alternaria* constituting a strongly supported group. *Lophiostoma* was phylogenetically distant from both the Clade I and Alternaria–Phoma–Paraphoma groups in Clade II.

### 3.3 Distribution and species diversity of DSE dominant species

**Figure 4:**
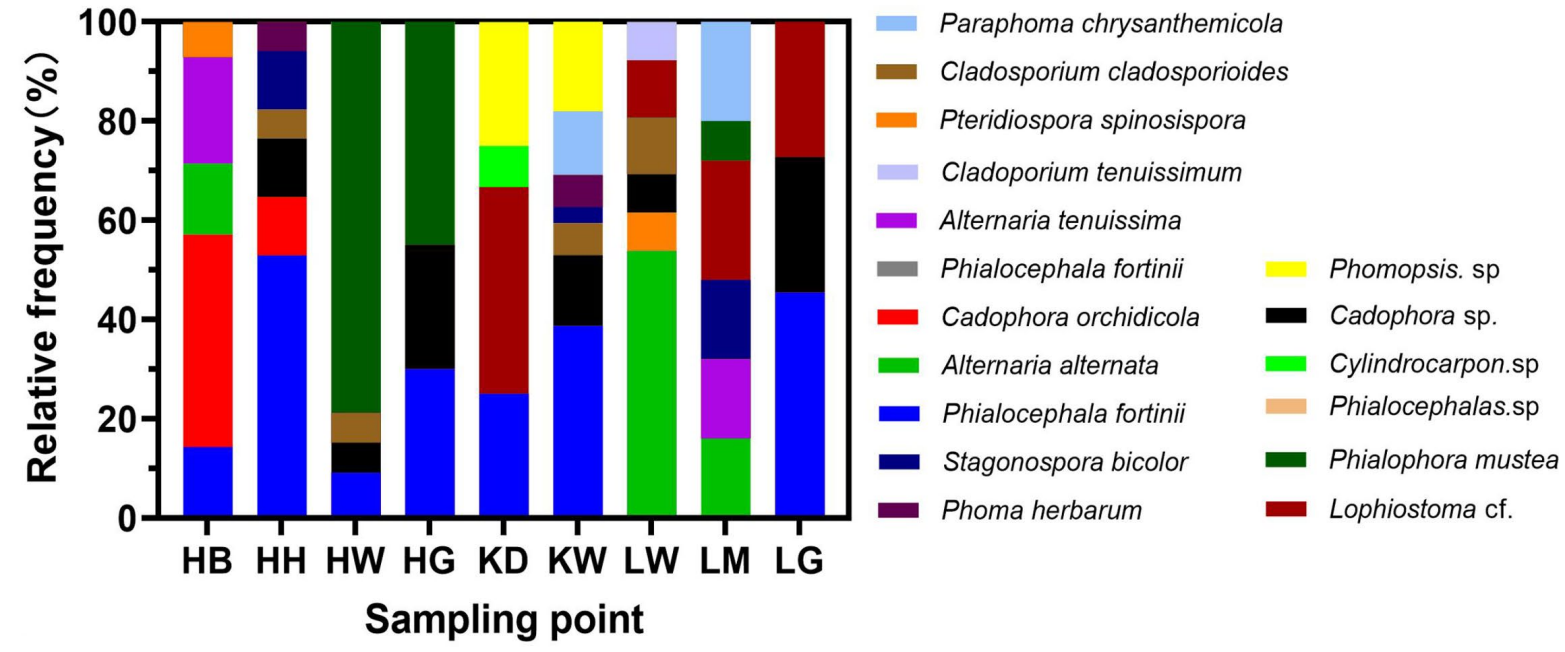
Genus-level relative frequency of DSE fungi associated with *U. pumila* roots across nine sampling areas.

Across the nine sampling areas, 16 DSE taxa were isolated (Figure 4). In HB, five species were identified: *Phialocephala fortinii*, *Cadophora orchidicola*, *Alternaria alternata*, *Alternaria tenuissima*, and *Pteridiospora spinosispora*, with *Cadophora orchidicola* being dominant (isolation frequency 42.85%). In HH, seven species were isolated, dominated by *Phialocephala fortinii* (52.94%). HW was dominated by *Phialophora muscea* (81.81%). HG yielded three species (*Phialocephala fortinii*, *Phialophora muscea*, *Cadophora* sp.), while KD yielded four species. In KW and LW, the dominant species were *Phomopsis* sp. (22.58%) and *Alternaria alternata* (53.84%), respectively. LM and LG each contained seven species, with LG dominated by a species exhibiting 45.45% frequency.

*Cylindrocarpon* sp. was found exclusively in KD, and *Cladosporium tenuissimum* only in LW. In contrast, *Cadophora* sp. was detected in seven areas (HB, HH, HW, HG, KW, LW, LG), indicating its widespread distribution in *U. pumila* roots in the northern regions studied.

**Figure 5:**
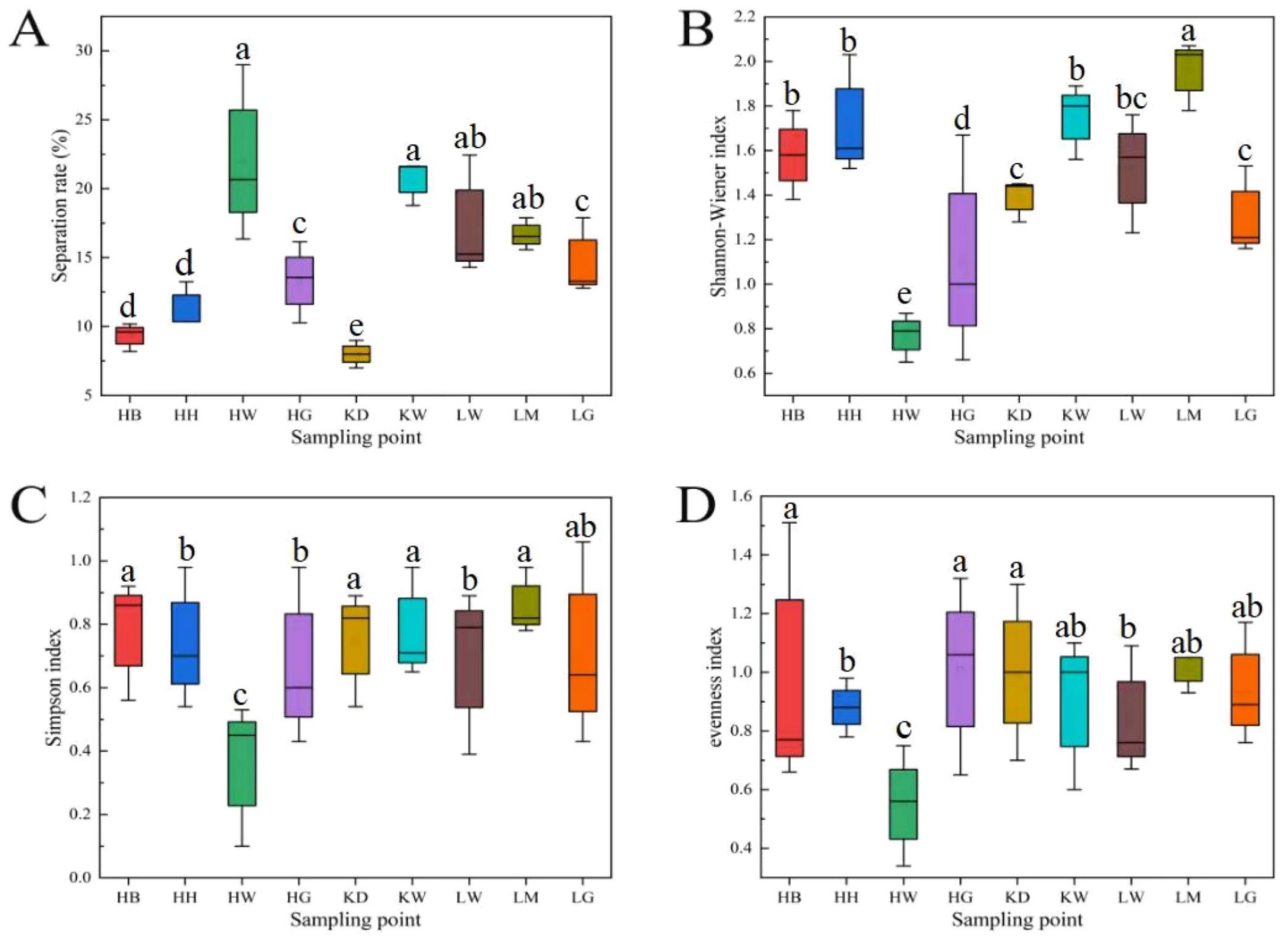
Boxplot of separation rates and diversity indices of DSE communities in the rhizosphere of *U. pumila*. Different lowercase letters indicate significant differences at the 0.05 level. **(A)** Isolation rate; **(B)** Shannon-Wiener index; **(C)** Simpson index; **(D)** Evenness index.

Significant differences were observed among the nine sampling areas for DSE isolation rate and diversity indices (Shannon-Wiener, Simpson, and Evenness) (Figure 5). The HW area exhibited the highest isolation rate (22%), which was significantly greater than that of HB, HH, HG, KD, and LG (*p* < 0.05). The LM area had the highest Shannon-Wiener and Simpson indices, being 2.55 times higher than the lowest value observed in HW and significantly higher than all other areas (*p* < 0.05). The Evenness index was highest in HB and lowest in HW, with both values differing significantly from other sampling sites (*p* < 0.05).

### 3.4 Similarity of DSE microbiota composition across sampling areas

DSE community composition varied markedly among sampling areas, as indicated by pairwise similarity coefficients (Table 2). HW and HG shared the highest similarity (coefficient = 0.428). Similarity coefficients among other sampling areas were all below 0.33, indicating low community overlap. Specifically, similarity coefficients between HB–KW, HB–KD, HW–KD, and HG–KD were all 0, suggesting that DSE species isolated under culture conditions did not overlap across these site pairs.

**Table 2:** Sørensen similarity coefficients of DSE microbiota in the rhizosphere of *Ulmus pumila* across sampling areas.

Sampling site	HB	HH	HW	HG	KD	KW	LW	LM	LG
HB									
HH	0.167								
HW	0.111	0.182							
HG	0.125	0.200	0.428						
KD	0	0.091	0	0					
KW	0	0.286	0.182	0.100	0.182				
LW	0.182	0.231	0.200	0.111	0.100	0.154			
LM	0.167	0.142	0.091	0.1	0.182	0.214	0.154		
LG	0.111	0.273	0.250	0.286	0.125	0.182	0.100	0.182	

### 3.5 Differences in soil nutrients across sampling areas

**Figure 6:**
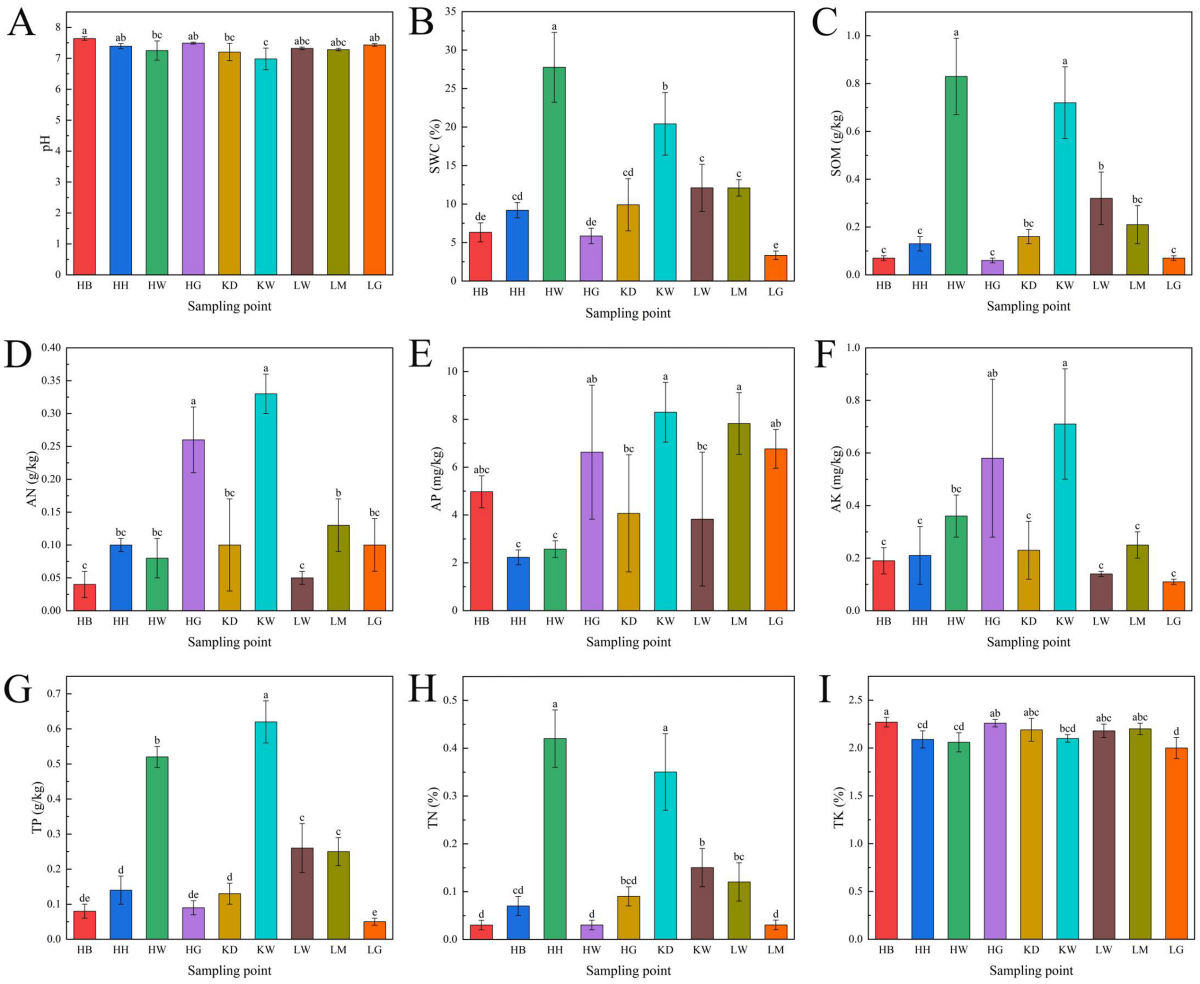
Physical and chemical properties of soil at the sampling site. Soil parameters: pH = soil acidity or alkalinity, SWC soil moisture content, AN = soil alkali-soluble nitrogen, AP = soil available phosphorus, AK soil available potassium, TP = soil total phosphorus, TN soil total nitrogen, SOM soil organic matter, TK = soil total potassium, the same below. Different lowercase letters indicate significant differences at the 0.05 level. **(A)** pH; **(B)** Soil moisture content (SWC); **(C)** Soil organic matter (SOM); **(D)** Soil alkali-soluble nitrogen (AN); **(E)** Soil available phosphorus (AP); **(F)** Soil available potassium (AK); **(G)** Soil total phosphorus (TP); **(H)** Soil total nitrogen (TN); **(I)** Soil total potassium (TK). Different lowercase letters indicate significant differences at the 0.05 level.

Significant differences (*p* < 0.05) were observed in the rhizosphere soil properties of *U. pumila* across the nine sampling plots (Figure 6). Soil pH ranged from 6.98 to 7.64, indicating weakly alkaline conditions in all plots except KW. SMC varied widely from 3.34 to 27.76%, with HW and KW exhibiting notably higher SMC than the other plots. SOM was lowest in HG (0.06 g/kg) and highest in HW (0.83 g/kg). Key nutrient concentrations also displayed substantial variation: alkali-hydrolyzable nitrogen (AN) ranged from 0.04 ± 0.02 to 0.33 ± 0.03 g/kg, available phosphorus (AP) from 2.23 ± 0.31 to 8.30 ± 1.25 mg/kg, and available potassium (AK) from 0.11 ± 0.01 to 0.71 ± 0.21 mg/kg. Among total nutrients, TP was highest in KW (0.62 ± 0.06 g/kg), more than 12-fold greater than the lowest value in LG (0.05 ± 0.01 g/kg). TN ranged from 0.03 ± 0.01% to 0.42 ± 0.05%, and total potassium (TK) was highest in HG (2.27 ± 0.05%), 1.14 times greater than the lowest value.

**Figure 7:**
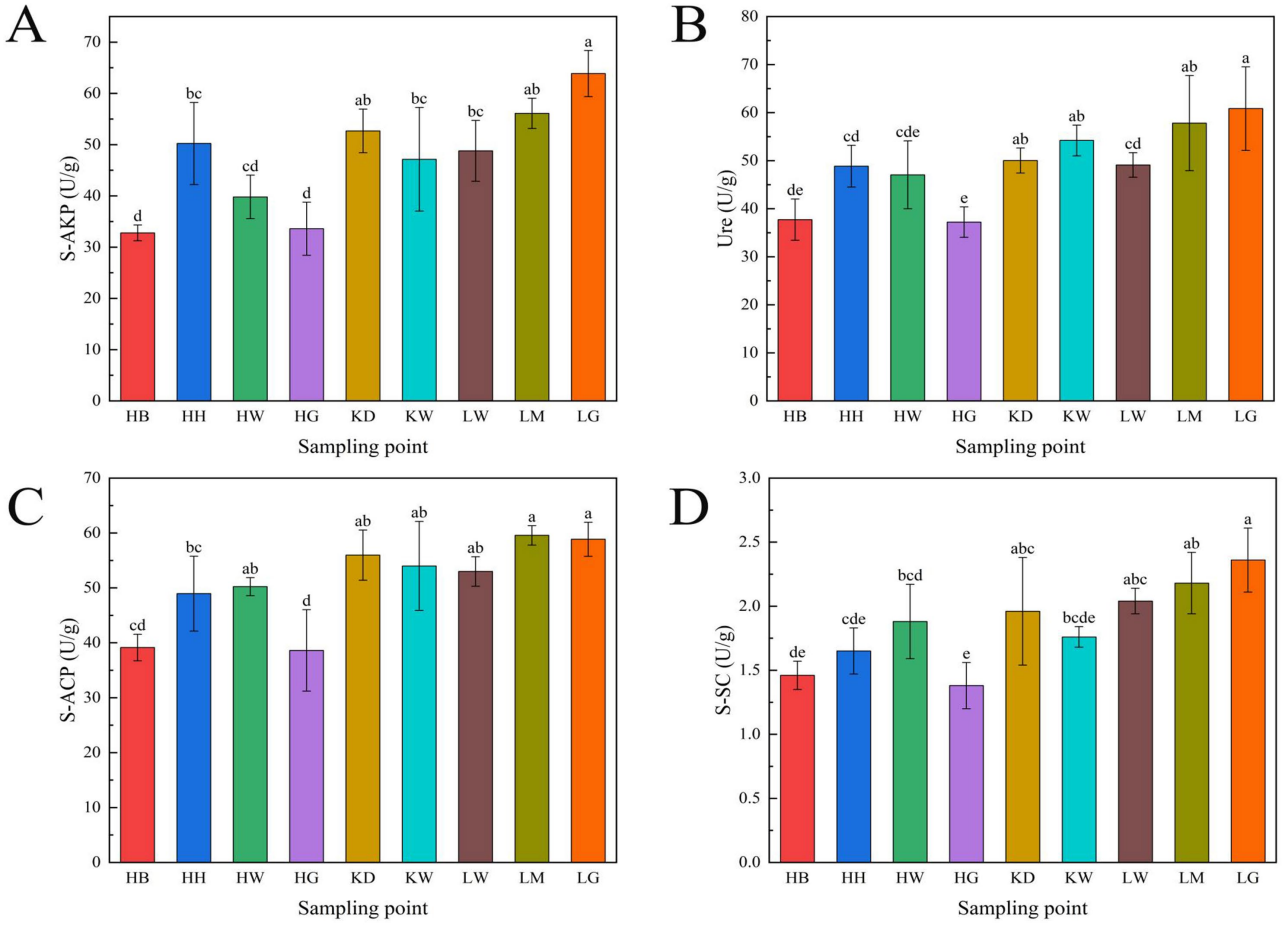
Soil enzyme activity at the sampling site. **(A)** Soil alkaline phosphatase activity (S-AKP); **(B)** Urease activity (Ure); **(C)** Soil acid phosphatase activity (S-ACP); **(D)** Soil sucrase activity (S-SC). S-SC = soil sucrase activity, Ure = urease, S-ACP = soil acid phosphatase activity, S-AKP soil alkaline phosphatase activity. Different lowercase letters indicate significant differences at the 0.05 level.

As shown in Figure 7, the activities of sucrase (S-SC), urease (Ure), acid phosphatase (S-ACP), and alkaline phosphatase (S-AKP) in the rhizosphere ranged from 1.38 to 2.36 U/g, 37.22 to 60.83 U/g, 38.61 to 59.56 U/g, and 32.78 to 63.86 U/g, respectively. Maximum enzyme activities were observed as follows: S-SC 2.36 ± 0.25 U/g (LG), Ure 60.83 ± 8.70 U/g (LG), S-ACP 59.56 ± 1.77 U/g (LM), and S-AKP 63.86 ± 4.50 U/g (LG). Soil sucrase activity was lowest in HG (1.38 ± 0.18 U/g), and urease activity in HG represented only 61.19% of the maximum. Acid phosphatase and alkaline phosphatase activities were lowest in HB, at 39.14 ± 2.41 U/g and 32.78 ± 1.53 U/g, respectively.

### 3.6 Relationships between soil nutrients, DSE isolation rate, and community diversity

**Figure 8:**
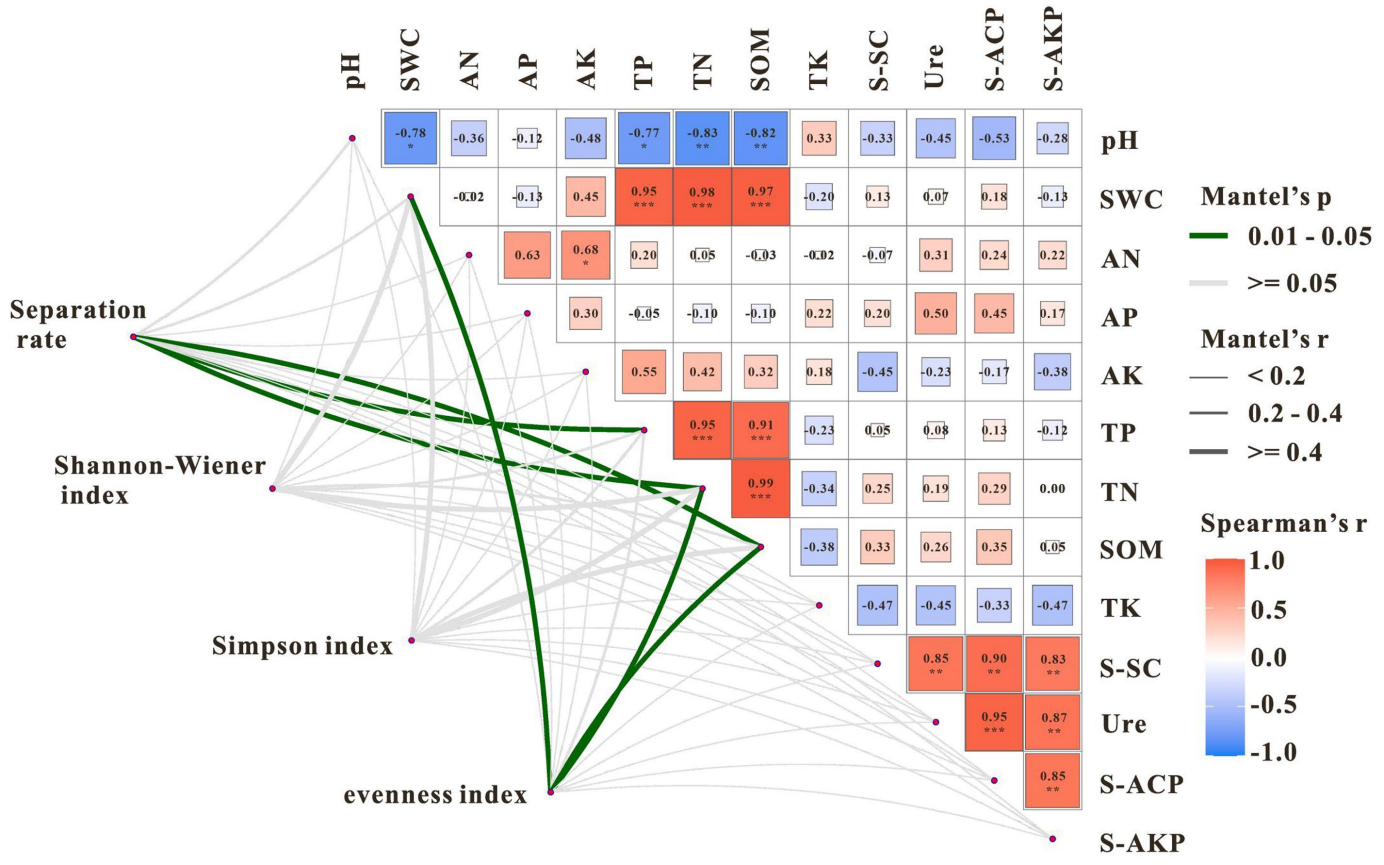
Mantel analysis of soil enzyme activity, physicochemical properties, isolation rate, and diversity indicators of DSE microbial community. *p < 0.05; **p < 0.01; ***p < 0.001.

Soil SMC was significantly positively correlated with TP, TN, and SOM (*p* < 0.001), with correlation coefficients of 0.95, 0.98, and 0.97, respectively (Figure 8). TP was significantly positively correlated with TN and SOM, and TN and SOM were also significantly positively correlated (*p* < 0.001). Sucrase (S-SC) was significantly positively correlated with urease (Ure), acid phosphatase (S-ACP), and alkaline phosphatase (S-AKP). Urease was significantly positively correlated with S-AKP, and S-ACP and S-AKP were also significantly positively correlated (*p* < 0.01). Correlation coefficients between pH and TN and SOM were −0.83** and −0.82**, respectively, and both were significantly positively correlated with SMC and TP (*p* < 0.05). Soil TP, TN, and SOM significantly influenced DSE isolation rate in the rhizosphere of *U. pumila*, whereas SMC, TN, and SOM were key indicators affecting the evenness of DSE communities.

**Figure 9:**
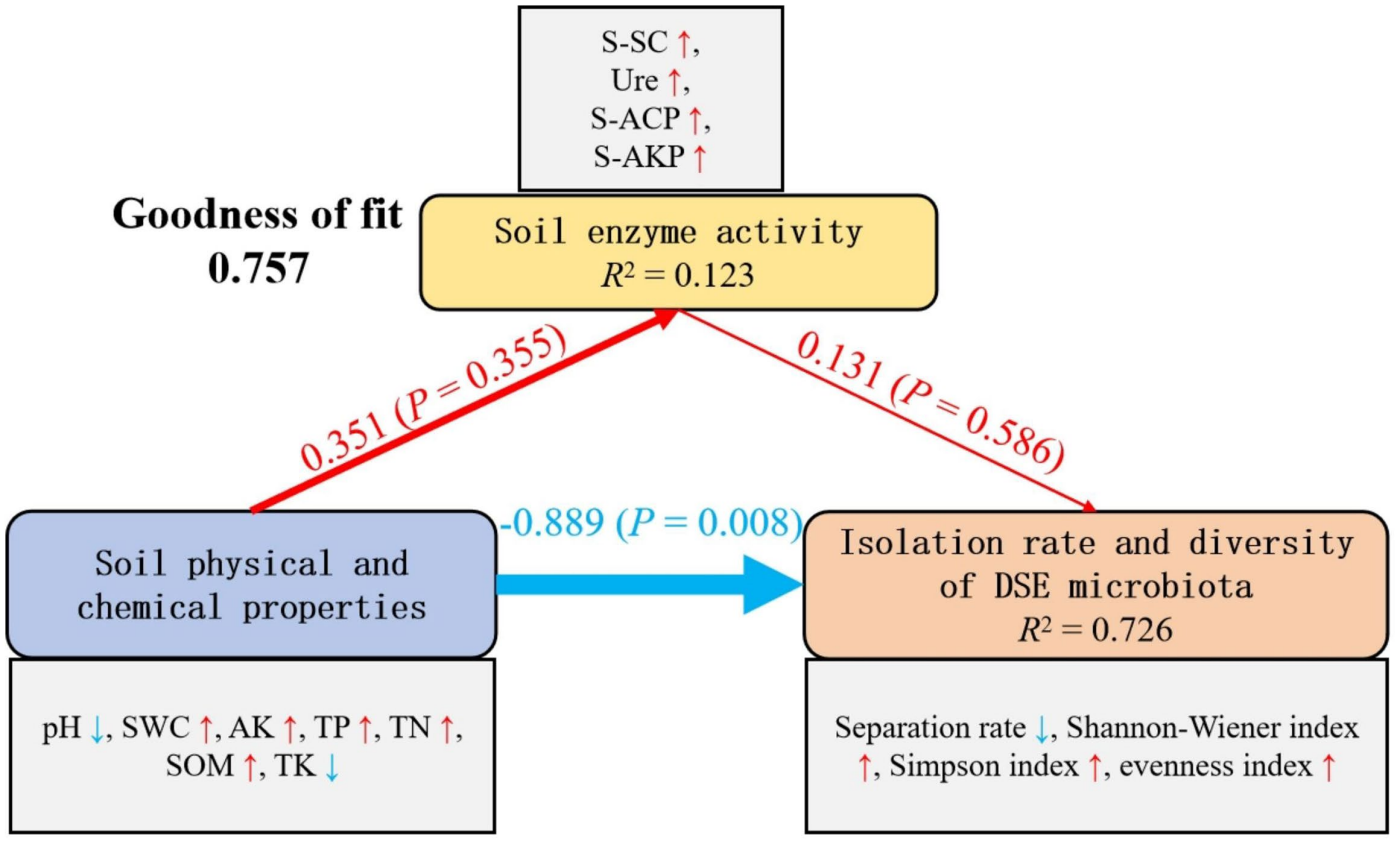
Partial least squares path modeling (PLS-PM) showing the influences of soil enzyme activity, soil physical and chemical properties, and isolation rate and diversity of DSE microbiota. Red lines indicate a positive path, and blue lines indicate a negative path. The thickness of the line indicates the magnitude of the path coefficient. The number on the red or blue line indicates the path coefficient.

Partial least squares path modeling (PLS-PM) revealed the differential effects of soil factors on DSE communities (Figure 9). The model exhibited a good fit (Goodness of fit = 0.757). Path analysis indicated a significant direct negative effect of soil physicochemical properties on both DSE isolation rate and community diversity. Although soil properties were positively correlated with enzyme activities, the resulting enzyme-mediated indirect effects were insufficient to counterbalance the strong direct negative effects. Consequently, the overall influence of soil physicochemical properties on DSE composition and diversity in *U. pumila* rhizosphere was negative.

## 4 Discussion

### 4.1 Isolation, species identification and diversity of root system DSE in *U. pumila*

Dark septate endophytes (DSE) are widely distributed root-associated fungi across various ecosystems, including forests, croplands, and sandy soils, and their beneficial effects on host plants have been well documented (Jin et al., 2018; Maulana et al., 2018). In this study, typical dark septate hyphae were observed within the root tissues of *U. pumila*, indicating that DSE can establish stable symbiotic associations with this species, consistent with the findings of Tian et al. (2022). Previous research by Knapp et al. (2012) suggested that endophytic fungal community composition may vary with the geographic location of the host plant, a hypothesis supported by subsequent studies (Liu et al., 2021; Pieterse et al., 2018; Zhao et al., 2015). Our results further demonstrate significant regional differences in DSE community composition in *U. pumila* roots, with dominant DSE taxa differing among sampling sites. This indicates that spatial heterogeneity in the growth environment influences DSE colonization patterns. Xie et al. (2017) proposed that higher community similarity indices between habitats correspond to more comparable biodiversity levels. Accordingly, the pronounced differences in DSE composition across the sampled areas reflect spatial environmental heterogeneity, supporting the close relationship between environmental variability and soil microbial community assembly. Such patterns may arise from environmental factors affecting host plant growth, which in turn shape the distribution and colonization of root-associated fungi. In this study, a total of 16 DSE strains belonging to 12 genera were isolated and purified. Based on ITS sequence analysis, 11 strains were identified to the species level, while the remaining five were resolved only to the genus level. Members of the genus *Alternaria* were frequently detected; these fungi are commonly associated with the roots of desert plants, appear to lack strict host specificity, and are likely adapted to arid environments (Han et al., 2021). Previous studies have reported that DSE can enhance host plant resilience to environmental stress (Han et al., 2021; Santos et al., 2021). In addition, some evidence suggests that endophytic fungal communities may exhibit host specificity (Sudová et al., 2020; Selim et al., 2012). However, the underlying mechanisms of DSE-host interactions remain largely unresolved. Future research should aim to elucidate the ecological functions of these DSE and their associations with host plants, particularly through inoculation experiments and functional assays to validate their growth-promoting effects and stress tolerance mechanisms.

Notably, among the DSE taxa isolated in this study, *Cadophora* sp. exhibited the broadest distribution, occurring in seven of the nine sampling areas (HB, HH, HW, HG, KW, LW, LG). This widespread occurrence suggests strong ecological adaptability to diverse soil conditions across the sandy lands of eastern Inner Mongolia. Previous studies have reported that *Cadophora* species are frequently associated with plants in harsh environments and can enhance stress tolerance via melanin deposition (Grünig et al., 2008; Day et al., 2012; Berthelot et al., 2016; Lopez et al., 2024). In this study, *Cadophora* sp. was present across sites with widely varying soil properties (SMC 3.34–27.76%, SOM 0.06–0.83 g/kg), indicating broad environmental tolerance rather than strict soil preference. This generalist strategy may confer competitive advantages in heterogeneous sandy ecosystems, enabling extensive colonization of *U. pumila* roots. Furthermore, *Cadophora* species have been reported to possess plant growth-promoting potential (Berthelot et al., 2016), and their widespread presence may enhance host environmental adaptability. Future studies should investigate the mechanisms underlying stress tolerance and growth-promotion by *Cadophora* sp. in *U. pumila* to better understand its ecological role in sandy land ecosystems.

### 4.2 Relationship between DSE colonization of *U. pumila* roots and rhizosphere soil nutrients

Soil serves as the essential substrate for plant growth, and variations in soil properties directly influence plant development and microbial community composition, thereby affecting the stability of sandy ecosystems (He et al., 2022; Sinsabaugh et al., 2015; Zhou et al., 2020). In this study, alkali-hydrolyzable nitrogen (AN) ranged from 0.04 to 0.33 g/kg, with HG and LW exceeding 0.30 g/kg (very high) and HB, HW, and KW below 0.10 g/kg (low). The rhizosphere soils of *U. pumila* in central and eastern Inner Mongolia were generally weakly alkaline (pH 6.98–7.64), with very low SOM (0.06–0.83 g/kg) and TN (0.03–0.42%), reflecting the poor nutrient retention capacity and limited microbial activity characteristic of sandy soils. The region’s drought conditions (SMC, ranging from 3.34 to 27.76%) may further impede OM decomposition and accumulation, resulting in low nutrient cycling efficiency.

Comparison of the nine sampling plots indicated that HW and KW exhibited high SMC (27.76 and 20.42%), along with elevated TN (0.42 and 0.35%) and SOM (0.83 and 0.72 g/kg), consistent with the general principle that higher soil moisture promotes OM accumulation. The peak available phosphorus (AP) in KW (8.30 mg/kg) may reflect enhanced phosphorus mobilization under increased water availability. Conversely, the extreme drought site LG (SMC = 3.34%) exhibited the lowest total potassium (TK, 2.00%), supporting the notion that drought exacerbates potassium leaching. Interestingly, AP in LG (6.77 mg/kg) exceeded that of most other plots, possibly due to acidic exudates released by *U. pumila* roots promoting the solubilization of insoluble phosphorus.

Soil enzymes, which are predominantly derived from microbial and plant root exudates, provide indicators of biochemical activity and soil fertility (Renella et al., 2006; Xiao et al., 2018). In this study, enzyme activities generally followed the pattern S-ACP > Ure > S-AKP > S-SC. The low S-SC values suggest limited sucrose hydrolysis capacity in the rhizosphere, indicating low organic carbon turnover and limited energy availability for soil biota. Meanwhile, Ure and phosphatase activities exceeded ecological thresholds, suggesting efficient utilization of nitrogen and phosphorus. Acid phosphatase activity (S-ACP) was notably higher than alkaline phosphatase (S-AKP), likely reflecting the preferential hydrolysis of organic phosphorus in weakly alkaline soils. This is consistent with previous reports indicating that acid phosphatase predominantly hydrolyzes organic phosphorus compounds, whereas alkaline phosphatase contributes primarily to inorganic phosphorus release (Kumar et al., 2016).

Partial least squares path modeling (PLS-PM) was employed to assess the direct and indirect effects of soil factors on DSE communities (Figure 9). The model exhibited good fit (Goodness of fit = 0.757), indicating reliable representation of variable relationships. Soil physicochemical properties—including decreased pH, increased SMC, AK, TP, TN, and SOM, and decreased TK—had a positive but non-significant effect on enzyme activities (path coefficient = 0.351, *p* = 0.355). Enzyme activities, in turn, exerted a weak positive effect on DSE isolation rate and community diversity (path coefficient = 0.131, *p* = 0.586). Notably, soil physicochemical properties had a significant direct negative effect on DSE isolation rate and diversity (path coefficient = −0.889, *p* = 0.008), suggesting that nutrient enrichment and altered soil conditions may suppress certain DSE taxa through environmental filtering. This contrasts with the common expectation that higher nutrient availability promotes microbial diversity, indicating that DSE in these habitats may be adapted to nutrient-poor conditions. Overall, soil physicochemical properties appear to constrain DSE community structure via direct negative effects, while enzyme-mediated indirect effects are insufficient to mitigate this influence, highlighting the dominant role of environmental stress in shaping DSE communities in sandy ecosystems. This study revealed that the spatial heterogeneity of DSE communities was primarily driven by environmental filtering, and the widespread distribution of Cadophora sp. reflected its broad ecological niche. The positive effects of TP, TN, and SOM on DSE colonization indicate that nutrient availability is a key factor limiting microbial colonization in nutrient-poor sandy land ecosystems. These findings provide new insights into the assembly mechanisms of rhizosphere microbial communities in sandy ecosystems and offer a theoretical basis for the conservation of *U. pumila* and the ecological restoration of sandy lands.

### 4.3 Limitations and future perspectives

This study has several limitations. First, culture-dependent methods may underestimate the true diversity of DSE fungi, as slow-growing or unculturable species are likely to be missed. Second, single-time sampling captures only the community structure during the peak growing season, precluding analysis of seasonal dynamics. Third, molecular identification based solely on ITS sequences provides taxonomic resolution but limited insight into ecological function.

Future research should incorporate inoculation experiments with key DSE strains to validate their growth-promoting and stress tolerance effects on *U. pumila*. Additionally, metagenomic and functional analyses could be employed to elucidate the metabolic potential and environmental adaptation mechanisms of DSE communities, thereby providing a more comprehensive understanding of their ecological roles in sandy land ecosystems.

## 5 Conclusion

In this study, a total of 200 dark septate endophyte (DSE) strains were isolated from nine sampling sites and classified into 16 species across 12 genera based on morphological and molecular identification. The composition of DSE communities in the rhizosphere of *Ulmus pumila* varied significantly among plots, with similarity coefficients between plots all below 0.33, reflecting substantial spatial heterogeneity. Rhizosphere soils were weakly alkaline and contained low levels of OM. Total soil potassium (TK) significantly suppressed the activities of sucrase (S-SC), urease (Ure), and phosphatases (*p* < 0.05) through ion antagonism, representing a key factor limiting nutrient cycling efficiency. TP content exhibited a moderate correlation with soil enzyme activities. Furthermore, TP, TN, and SOM significantly influenced the DSE isolation rate in *U. pumila* rhizospheres, whereas SMC, TN, and SOM were critical determinants of the Evenness index of DSE communities. Among the sampling sites, Saihanwula, Wulanmaodu, and Wubulbaolige—characterized by mountain black soil and black calcic soil—exhibited superior soil nutrient profiles. The Saihanwula site, dominated by mountain black soil, performed best, achieving the highest DSE isolation rate of 22%. These findings indicate that soil type strongly influences DSE colonization and that targeted improvement of soil nutrient status can facilitate DSE establishment and growth. Overall, the results provide valuable theoretical insights into the interactions between climax plant communities and associated microorganisms in the central and eastern regions of Inner Mongolia, while also offering practical guidance for subsequent mycorrhizal research on *U. pumila* and strategies for sustainable ecosystem management.

## Data Availability

The datasets presented in this study can be found in online repositories. The names of the repository/repositories and accession number(s) can be found in the article/Supplementary material.

## Footnotes

1
https://www.ncbi.nlm.nih.gov
