# Correction: Study on dark septate endophytic (DSE) fungi and soil nutrients of *Ulmus pumila* L. in sandy land in eastern Inner Mongolia

**DOI:** 10.3389/fmicb.2026.1925015

**Published:** 2026-07-15

**Authors:** Yunxia Ma, Yuexin Zhang, Guang Yang, Yazhou Shao, Xiaolu Ma, Haibing Wang

**Affiliations:** 1 State Key Laboratory of Water Engineering Ecology and Environment in Arid Area, Inner Mongolia Agricultural University, Hohhot, China; 2 College of Desert Control Science and Engineering, Inner Mongolia Agricultural University, Hohhot, China; 3 Department of Ecology and Meteorology, College of Forestry, Inner Mongolia Agricultural University, Hohhot, China

**Keywords:** community structure, dark septate endophytic fungi, sandy land, soil nutrients, *Ulmus pumila* L.

There was an error in the **Funding** statement as published.

The funding statement originally read: “Science and Technology Support Project of the Department of Science and Technology of Inner Mongolia (grant number KJZC-2025).”

The correct **Funding** statement reads: “2025 Science and Technology Support Project of State Key Laboratory of Water Engineering Ecology and Environment in Arid Area (grant number 2025GJCXPT01020).”

The original version of this article has been updated.

