# Supplementary material for: Study on dark septate endophytic (DSE) fungi and soil nutrients of Ulmus pumila L. in sandy land in eastern Inner Mongolia
Source: Front Microbiol. 2026 Apr 13;17:1782229. doi: 10.3389/fmicb.2026.1782229 (PMC13112535; doi:10.3389/fmicb.2026.1782229)
Supplement: Supplementary file 1 [file Table_1.DOC]

T**able.S1** Sample collection information.

| **Sampling site** | **Abbreviation** | **Latitude** | **Longitude** | **Altitude(m)** | **Soil properties** | **Annual temperature(**℃) | **Average annual precipitation(mm)** |
| --- | --- | --- | --- | --- | --- | --- | --- |
| Baiyin Aobao | HB | N43°31′52″ | E117°13′58″ | 1361.82 | sandy soil | 4 | 320 |
| Huamugou | HH | N42°42′46″ | E117°20′4″ | 1371.95 | gray forest soil | 3.5 | 420 |
| Saihanwula | HW | N44°13′4″ | E118°43′4″ | 1150.41 | mountain black soil | 2 | 358 |
| Sangen Dalai | HG | N42°42′44″ | E115°55′53″ | 1307.25 | chestnut soil | 1.5 | 365 |
| Daqinggou | KD | N42°47′55″ | E122°10′24″ | 248.21 | sandy loam soil | 5.6 | 450 |
| Wulan Maodu | KW | N46°17′49″ | E120°47′19″ | 575.97 | chernozem | 1 | 380 |
| Wubulbaolige | LW | N48°4′40″ | E119°40′22″ | 791.69 | chernozem | -2 | 280 |
| Manzhouli | LM | N49°34′24″ | E117°25′17″ | 647.59 | chestnut soil | 0.7 | 300 |
| Hailar | LG | N49°12′45″ | E119°42′16″ | 618.06 | chestnut soil | -2 | 370 |

**Table.S2 Morphological characteristics of DSE in the rhizosphere of *U. pumila***

| **Strain** | **Colony color** | | **Colony characteristics** | **Mycelium** | **Secretion** | **Spore** |
| --- | --- | --- | --- | --- | --- | --- |
|  | **Front** | **Back side** |  |  |  |  |
| BYAB3 | black | black | surface roughness with fuzz; bulge | rough, clearly separated | a small amount of white secretion | clustered shape |
| BYAB4 | grayish black | black | surface roughness with fuzz; bulge | smooth, clearly separated | brown yellow secretion, pigment seeps into the culture medium | none |
| HMG3 | dark green | grayish black | surface roughness with white fuzz; bulge | smooth, clearly separated | none | none |
| HLE1 | dark brown | dark brown | surface roughness with fuzz; Felt like protrusions with grooved patterns | smooth, clearly spaced, with small spacing between compartments | none | none |
| WBEBLG7 | grayish green | grayish green | surface roughness with fuzz; Cotton like protrusion | smooth, clearly spaced, with small spacing between compartments | a small amount of white secretion | none |
| HMG4 | dark green | black | surface roughness, powdery bumps | smooth, not clearly separated | black secretion | Clustered shape |
| DQG5 | dark green | dark green | the surface is concentric, rough, and has white fluffy felt like protrusions | smooth, clearly separated | green secretion and pigment infiltration into the culture medium | none |
| DQG3 | grayish black | black | the surface is concentric and circular, with radiating edges, rough, and cotton like protrusions | smooth, clearly separated, with thick mycelium | none | none |
| HLE3 | dark green | black | the surface is rough with obvious residual secretions, and the edges are serrated with fuzzy bumps | smooth, clearly separated | there is white secretion on the surface | none |
| DQG2 | dark brown | black | surface roughness, radiating edges, cotton like protrusions | rough, clearly separated | none | disperse |
| SHWL6 | dark green | black | the surface is concentric and ring-shaped, with radiating edges, rough, cotton like protrusions, and grooved patterns | smooth, clearly separated | none | none |
| SGDL2 | black | black | surface roughness, cotton like protrusions, and radiating edges | smooth, clearly separated | none | disperse |
| WLMD6 | dark green | dark green | the surface is rough, with brown cotton like fluff in the center of the colony | smooth, not clearly separated | none | none |
| DQG4 | brown | black | the surface is rough with felt like hairs, and the aged mycelium on the surface appears white | smooth, not clearly separated | none | none |
| WBEBLG3 | green | dark green | smooth surface, felt like protrusions, and radiating edges | smooth, not clearly separated | none | none |
| MZL7 | brown | black | surface roughness, concentric ring shape, felt like protrusion, with pigments infiltrating into the culture medium | smooth, not clearly separated | none | none |
